# Centering context when characterizing food environments: the potential of participatory mapping to inform food environment research

**DOI:** 10.3389/fnut.2024.1324102

**Published:** 2024-02-21

**Authors:** Shauna Downs, Swetha Manohar, Wiktoria Staromiejska, Chanvuthy Keo, Sophea Say, Nyda Chhinh, Jessica Fanzo, Serey Sok

**Affiliations:** ^1^Department of Health Behavior, Society and Policy, Rutgers School of Public Health, Newark, NJ, United States; ^2^Global Food Ethics Policy Program, School of Advanced International Studies, Washington, DC, United States; ^3^Nutrition, Diets and Health Unit, International Food Policy Research Institute, Washington, DC, United States; ^4^Faculty of Social Science and Humanities, Royal University of Phnom Penh, Russian Federation Boulevard, Khan Toul Kork, Cambodia; ^5^Department of Tourism, Royal University of Phnom Penh, Phnom Penh, Cambodia; ^6^Department of Economic Development, Faculty of Development Studies, Royal University of Phnom Penh, Russian Federation Boulevard, Khan Toul Kork, Cambodia; ^7^Columbia’s Climate School, Columbia University, New York, NY, United States; ^8^Research Office, Royal University of Phnom Penh, Russian Federation Boulevard, Khan Toul Kork, Cambodia

**Keywords:** food environment, participatory mapping, Mekong River, Tonle sap lake, focus group discussion

## Abstract

Food environments are a critical place within the food system to implement interventions aimed at enabling sustainable diets. In this perspective article, we argue for the need for food environment research to more comprehensively examine the different types of food environments that people access within their communities to ensure that interventions and programs are better aligned with people’s lived experiences. We highlight the potential ways in which participatory mapping (PM) can be leveraged to better design food environment research by: (1) identifying the different food environment types that are accessed within a given community; (2) providing insight into the timing for data collection; (3) informing the prioritization of where to conduct food environment assessments; and (4) highlighting the dynamism of food environments over time (e.g., across a given day or across seasons). We provide a case study example of the application of PM and the lessons learned from it in Cambodia. By conceptualizing food environments in a more comprehensive way, from the perspective of the people living within a given community, we will be able to measure food environments in a way that more closely aligns with people’s lived experiences.

## Introduction

Food environments are a critical place within the food system to implement interventions aimed at enabling healthy and sustainable diets. While several definitions (1–4) for the food environment exist, we define the food environment as “*the consumer interface with the food system that encompasses the availability, affordability, convenience, promotion and quality, and sustainability of foods*” (5). Food environments in low- and middle-income countries (LMICs) are much more multifaceted than what we typically observe in high-income countries (HICs). While in HICs we often characterize food environments as the built food environments that people have access to (6), in LMICs many people access diverse food environments (5). This includes wild, cultivated, built (formal and informal) environments (5), as well as kin and community and supplemental food assistance (7) (see Box 1). These different food environment types subsequently influence the foods that are acquired, purchased, and consumed from them which has implications for diet and nutrition outcomes, as well as interventions aimed at improving those outcomes.

BOX 1 **Overview of food environment types [Adapted from: (5), (7)].**

**Natural food environments:** Natural food environments include both wild (e.g., water bodies, wetlands, forests, jungles, etc.) and cultivated (e.g., fields, gardens, pastures, etc.) environments where people access food for own consumption.

**Built food environments:** The built environment includes both informal and formal food market food environments. Informal market food environments include wet markets, mobile vendors, kiosks, etc., whereas formal markets include supermarkets, restaurants, and other formal retailers. The same vendor types (e.g., street vendors) could be informal or formal, depending on the context. Formal vendors are regulated in some way, whereas informal vendors are not.

**Kin and community:** Kin and community (or social networks within communities) includes gift or exchange of food from friends, neighbors, or other community members, food assistance provided by charities, food obtained from social or cultural gatherings as well as food remittances.

**Supplemental food assistance and aid:** Supplemental food assistance and aid is food provided through government or non-governmental food provision systems.

Access to different food environment types has been found to be associated with differences in food security (8), dietary intakes (1), and nutrition outcomes (9). In most LMIC contexts, foods are procured by interacting with natural food environments through means of hunting, fishing, and foraging in wild settings, and growing foods in cultivated settings (5, 10). This can also be true in HIC, particularly within indigenous communities (11, 12). However, the majority of food environment research in HICs focuses on the built environment with little recognition of food access from the natural environment. While we focus this article on the need to recognize and incorporate the heterogenous food environment types (especially the natural food environment) into food environment research in LMICs, this holistic approach has implications for HIC settings as well. For example, there has been a recent resurgence of growing food for household consumption in HICs, partly related to the COVID-19 pandemic (13). Thus, it is important that food environment research in both HICs and LMICs examine the variety of food access points of populations of interest.

While access to the natural food environment has been attributed to increased access to nutrient-rich foods and dietary diversity, access to modern retail has been shown to have more mixed influences on diets and nutrition (14). In the Solomon Islands, having access to wild, cultivated, and kin and community environments was found to be associated with improved fruit and vegetable acquisition, whereas access to formal retail environments was associated with acquisition of less fruit and vegetable yet more ultra-processed foods (7). In urban informal settlements in Kenya, a majority of kiosks and hawkers have been characterized as predominantly selling fried starches and sweets/confectionary, respectively (15). Other evidence from Central Province, Kenya, reveals acquiring foods at a supermarket vs. any other retailers (self-service stores and kiosks) was associated with a higher body mass index (BMI) and probability of overweight among adults (16). In children, supermarket food purchases were associated with improvements in child growth, without any contributions to obesity (17), demonstrating both positive and negative impacts that formal retailers can have on diets and nutrition.

There is a growing recognition of the need to better measure food environments in order to inform the design and implementation of interventions to promote healthy and sustainable diets that are more closely tailored to their context. In this perspective article, we argue for the need to include the natural environment, as well as any other food access points [e.g., kin and community (7)] in food environment assessments conducted in LMICs in particular, but even in HIC contexts where food environments may be diverse [e.g., indigenous communities (10)]. Often, the heterogeneity of food access points might not always be immediately apparent. As such, we further highlight the potential of leveraging participatory mapping (PM) as a methodology to better, and inclusively, design studies that aim to characterize food environments in a more comprehensive way. We provide a case study example of its application and the lessons learned from it in Cambodia.

## Integrating participatory mapping into food environment studies

### Participatory mapping

Participatory mapping is a focused ethnographic method that encourages participants to collaborate in drawing a map of their local community and discussing the importance of the different landmarks and assets depicted within it (18). It is a relatively quick method, taking approximately 90 min to employ, and can be conducted as part of focus group discussions (FGD). The goal of PM is “to make visible the relationship between a place and local communities” by creating a visual representation of how members of a given community perceive it (19). PM can include various tools including sketch mapping, transect mapping, Geographical Information Systems (GIS) mapping, remote sensing images, among others (19, 20). It has historically been used to map community assets such as schools, health centers, and other key landmarks to inform development initiatives. In the context of food environment research, PM can be used to generate a map of the food access points within a given community, by those living within it. By working together as a group to map food access points, and to discuss their importance for the community, the FGDs conducted as part of the PM allow for community members to work together to establish a common understanding of their community food environments. It empowers individuals to contribute their knowledge and perspectives to the mapping process based on interactions with their own surroundings and to discuss the community’s experiences related to food availability, affordability, quality, etc. The PM can be used as part of a broader participatory research approach or could be used as a standalone participatory method. While the maps produced as part of the PM process can vary in terms of quality, they can be used as an important starting point for guiding discussion among the group of participants and generating key contextual information about the community’s food environment that “fills in the gaps” of traditional GIS mapping techniques.

### Application of participatory mapping to food environment research

Participatory mapping can make a unique and important contribution to the design of food environment research, including: (1) identifying the different food environment types that are accessed within a given community by centering the community’s lived experience; (2) providing insight into the timing for data collection within the informal sector in particular; (3) informing sampling for food environment assessments; and (4) highlighting the dynamism of food environments over time (e.g., across a given day or across seasons).

#### Food environment access points

First, PM allows for the identification of the different food access points—the places and spaces where people access food—consumers in a given community acquire food from. It has been used by members of our team in Myanmar, Kenya, and Cambodia (21, 22). When trying to characterize food environments, understanding all the food access points is critical, particularly in countries that are experiencing food environment transitions (23) and for communities that heavily rely on natural food environments for their livelihoods, food security, sociocultural traditions, and nutrition (24, 25). Characterizing participants’ perceptions of personal and external food environment dimensions (e.g., food availability/accessibility, food prices/affordability, convenience, promotion and quality, and sustainability) (Box 2) in the different food access points that they interface with is also critical. These dimensions apply to all food environment types, with the exception of food prices/affordability which are not directly captured in wild and cultivated environments. In the FGDs, participants first identify the different spaces where they access food and subsequently discuss how they access those spaces (see Supplementary material A to review an example FGD guide). While the built and wild food environments tend to be communal spaces, the cultivated food environment often includes individual spaces such as home gardens or household plots of land. In these cases, the discussion focuses on the community experience with cultivated food access points rather than individual plots of land, gardens, etc.

BOX 2 **Defining food environment dimensions captured in participatory mapping [adapted from: Downs et al. (5) and Turner et al. (4)].**

**Availability/accessibility**: Availability refers to whether a food item is present within a given physical range (external food environment) and accessibility refers to physical distance, mobility, mode of transport, and individual activity spaces (personal food environment).

**Food prices/Affordability**: The prices of food items relative to other foods or to a defined income standard (e.g., % of median income or % of poverty line).

**Convenience:** Time cost of obtaining, preparing, and consuming a food item.

**Promotion:** How a food item is presented, marketed, promoted, and front-of-pack labeling which is designed to influence the desirability of food.

**Quality:** External characteristics of food including its freshness, integrity, safety, nutrient and phytochemical profiles, and objective sensory attributes.

**Sustainability:** The environmental and social impact associated with the food item.

#### Timing of data collection

Informal markets and vendors often vary in terms of where, when, and how much they sell food. As such, the ways in which consumers interface with this informal food environment may also vary. For this reason, it is important that food environment data collection be informed by consumers’ knowledge about how the informal sector operates in their given community. Moreover, mobile vendors are difficult to capture in food environment research and could be easily missed depending on the timing of data collection. Since the COVID-19 pandemic, mobile vendors have emerged as being critical in terms of increasing food access in some communities in LMICs (26, 27). PM can provide researchers with insights into the hours of operation of informal markets and the reliance on mobile vendors in a given community, which can subsequently inform their approach to data collection. PM can also inform the timing of food environment data collection based on local festivals or holidays (28) or how consumers strategize the purchase of different foods (29) which can influence food availability and prices, among other food environment dimensions.

#### Prioritization of where to conduct food environment assessments

Participatory mapping can help to identify which food access points should be prioritized for conducting food environment assessments as well as the food outlets within them (for built environment) that should be targeted. For example, open-air traditional markets have historically played an important role in providing fresh, nutritious, foods to rural and urban populations alike (30–32). However, there have been shifts in their importance in some settings in the aftermath of economic, COVID-19, and conflict shocks in some settings (33). The PM can help inform whether it makes sense to conduct food environment assessments in open-air markets, and which markets to focus data collection on based on participants’ discussion of the role of those markets in terms of their food access. Conducting PM can also provide insight into where to capture data to characterize different food environment dimensions (e.g., food prices/affordability). For example, food price data should be collected from the types of food outlets people primarily purchase their food from vs. outlets that may appear to be the dominant access points in the community. This may not be intuitive given that consumers make many trade-offs (e.g., convenience of purchasing near to home with higher food prices or purchasing food via digital apps rather than physically going to stores to purchase food) when deciding what food to acquire and from where.

#### Highlighting the dynamism of food environments over time (e.g., across a given day or across seasons)

Seasonal changes to food environments are evident in LMICs and HICs alike. For example, availability, accessibility, and affordability of foods acquired in rural and urban Malawi (34), urban India (35), and urban areas in the United States (36) is significantly impacted by seasons. Seasonality can also influence access to vendor types with certain vendors not being accessible during certain seasons, such as wet and dry (36, 37). However, much of the food environment literature has failed to take into account seasonal changes in food environments (4). PM can be practical in terms of gaining consumers’ perceptions of how their food environments shift across seasons in terms of the types of food environments they access and the dimensions with them. It can also help determine which food environment assessment, if any, should be conducted in different seasons (wet or dry season) to account for seasonal variation. This can help to optimize resources for food environment research.

In the next section, we provide a case study that demonstrates how PM can be leveraged to streamline food environment data collection in Cambodia.

## Case study: application of participatory mapping in Cambodia

As part of a larger project—A River in Transition: understanding the health and environmental sustainability of consumer food choice, local food environments and diets in riverine communities of the Lower Mekong Basin (LMB) —we conducted eight PM FGDs in four provinces (2 communes per province) along the Mekong River and the Tonle Sap Lake in Cambodia. Each focus group included 6–8 women consumers (total *n* = 59) living in the communes (administrative divisions of Cambodia are divided into province, district, commune, and village), given that they were the primary food shoppers in the study communities. The PM was designed to inform the food environment data collection approach in each of the communes for the project. Ethical approvals were obtained from the National Ethics Committee for Health Research in Cambodia and the Johns Hopkins University Homewood and Rutgers University Institutional Review Boards in the United States.

FGDs were conducted between February and March 2023 and were moderated by a senior researcher, with the help of an assistant. The FGD guide for the PM can be found in Supplementary Material A. In short, the FGD included the following topics: where people go to access food, foods purchased and from where, most commonly and infrequently used vendors, changes in access to markets/vendors over time, foods grown and foraged and from where, foods acquired and exchanged with neighbors and who consumes grown and foraged foods. Within each FGD, participants drew a map of their food environment (see Supplementary Material B for an example). The FGD took an average of 85 min to complete and were conducted in Khmer. As part of the FGD, the participants created maps of their communes with the different food environment types that they accessed. Table 1 provides a description of the participant characteristics. The FGDs were audio-recorded and subsequently translated to English and transcribed verbatim.

**TABLE 1:** Study participant characteristics (*N* = 59)*.

Sample characteristics	% (*n*)
**Age**	
19–24 years	11.9 (7)
25–34 years	33.9 (20)
35–44 years	23.7 (14)
45–55 years	15.3 (9)
>55 years	15.3 (9)
**Education**	
None	10.2 (6)
Primary, not completed	22.0 (13)
Primary, completed	22.0 (13)
Secondary, not completed	10.2 (6)
Secondary completed	30.5 (18)
Some higher education, no degree	5.1 (3)
**Primary occupation**	
Homemaker	64.5 (41)
Small business owner	6.8 (4)
Daily/casual labor	6.8 (4)
Full-time salaried worker	17.0 (10)
**Marital status**	
Single	6.8 (4)
Married	88.1 (52)
Widowed	5.1 (3)
Divorced/separated	0

*Eight focus group discussions were conducted with 6–8 participants in each discussion.

We used open coding to analyze the FGD transcripts using NVivo software [release 14.23.0 (13)]. Open codes were subsequently organized by key themes for each of the communes separately. We also examined key themes that cut across different communes. We present findings related to the ways in which leveraging PM in this study helped inform the food access points, the timing of data collection, the sampling for food environment assessments, and seasonal changes in food environments.

### Key findings related to food environment research implementation from participatory mapping

#### Food access points

The PM provided important insights into the places people in the 8 communes accessed food. Key themes related to food access points included: the importance of mobile vendors and “*family retail*” (e.g., small stores selling food often attached to people’s homes) for daily food purchases, the minor role of markets for food purchases in most communes, and the critical role of wild and cultivated food environments, as well as kin and community, for providing a safety net to ensure food security. These populations reported a heavy reliance on the wild and cultivated environment for their food. As one participant stated: “*We grow vegetables to just feed our household, avoiding dependence on the market*.” In addition, community members in 7 of the 8 communes also shared that they sell, trade and/or share food with other people in their communities. In one commune, a community member stated “*sometimes, I plant the vegetables that the other houses don’t, so we exchange*” indicating that cultivation of certain foods is at times strategically planned among households. These social networks were viewed as creating camaraderie among villagers: “*It is an easy life in our village. Some villages are not. Villagers here are kind. We love one another*.” Thus, if researchers were to solely capture dimensions of the built food environment in this context, they would miss critical food access points that provide access to nutrient-rich foods such as animal-source foods, leafy greens and other vegetables, as well as fruit.

Another key learning from the PM related to food access points was the shift away from accessing open-air markets and a heavy reliance on mobile vendors in several communities. More specifically, we found that in 6 of the 8 communes, the community members indicated that they rarely go to the nearest local markets to purchase foods. In most cases, these communities “*only buy from the family retail or the motorbike groceries*” selling a wide variety of food to meet their household’s needs. Lastly, while this project was being conducted in Cambodia, one of the study communes was situated near the border with Vietnam. In that community, focus group participants reported often accessing a nearby market in Vietnam.

#### Timing of data collection

The PM helped to inform the timing of data collection in the study communes. This was important given that some markets within the larger landscape of the built food environment changed significantly over the course of the day. In some cases, the early hours of the morning were when the market was in full operation, whereas in other markets it was in the evening. FGD participants described these as “*waxing*” markets, where their size varied with the time of the day. As one FGD participant stated: “*They only sell in the morning*…*if you are late, there is nothing else*.”

#### Prioritization of where to conduct food environment assessments

The findings from the PM related to identifying food access points also helped to inform which markets and vendors should be prioritized for the food environment assessments in each commune. For example, in communes where people reported not regularly accessing the nearest local markets, we did not conduct any food environment assessments at those markets. Instead, we focused on the food access points that consumers indicated that they purchased food from such as local small groceries and mobile vendors, allowing us to characterize the food environment with which the community most frequently interacts with. In some cases, these were brick and mortar food outlets, and in other cases, these were completely informal vendors.

#### Highlighting seasonal changes in food environments

**FIGURE 1:**
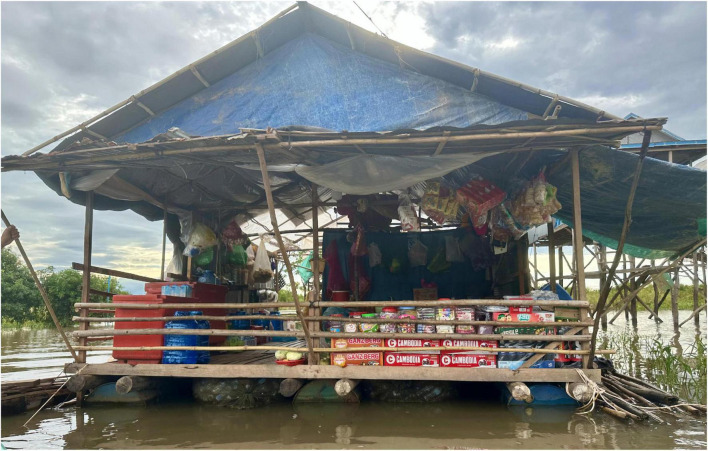
An example of a floating small grocery store in wet season.

The PM exercise also helped to inform the changes that consumers observe in their food environments across seasons. Key themes related to seasonal changes included variation in the number, and mode of travel (e.g., shifting from motorbike to boat) of mobile vendors, changes in food access from wild and cultivated environments, and the increase in the time and economic cost of accessing markets in the wet season. For example, in one of the communes included in our sample, the wet season changes the village landscape to a partially, or fully, floating village depending on the year (see Figure 1). This has important implications in terms of where and how people access food. For example, some of the mobile vendors that sell food by motorbike during the dry season shift toward selling food by boat during the wet season. However, the degree to which this occurs depends on the degree of flooding in the community each year. Moreover, in all the FGDs where community members noted the importance of mobile vendors, they indicated a decline in their availability in the wet season.

Other important aspects related to seasonal changes in food environment relate to the natural environment. In all the FGDs, participants mentioned substantial differences in the foods that they have access to during the dry as compared to the wet season. In particular, many of them also described the influence of more extreme temperatures, seasons shifting to different months, and a change in the availability and abundance of foods from the natural environment across seasons.

## Discussion

In this perspective, we highlight the role of using PM to provide insights into consumers’ food environments in a way that better informs approaches to food environment data collection. This case study from Cambodia helps to provide an example of its application to food environment work in a LMIC. However, we anticipate that PM can also be used to help inform food environment research in HICs as well, given that populations in HICs also access wild and cultivated spaces as evidenced by the paper by Coffin-Schmitt et al. included in this Research Topic (13).

While we argue that including PM as a formative, exploratory step to inform food environment data collection approaches can strengthen food environment research, there is a clear need for methods and data collection tools that are tailored to these diverse and dynamic food environments (5). There are several groups of researchers who are currently working toward designing food environment assessment tools that are more relevant to the food environments that consumers interface with in LMICs. PM can then help to identify which methods and tools would be most appropriate for measuring the food environment in a given context.

One of the key learnings of understanding food environments from the perspective of consumers using PM is their dynamism, which has implications for measuring them. For example, in the case of market mapping assessments, such as the assessment included in the USAID Advancing Nutrition Guidelines for Market-based Food Environment Assessments (38), the timing of the assessment would need to be aligned with the peak market days, and times within those days. Moreover, it is likely that conducting assessments across different seasons might be necessary in some settings to capture cross seasonal variation in food environment dimensions.

Participatory mapping can also be leveraged to identify which markets you might prioritize measurement of. For example, we found in the PM that we conducted in Cambodia that consumers were crossing the border to access food from a major market in Vietnam. Ideally, we would then conduct market mapping at that market as part of the food environment research; however, this requires additional research permits, IRB approvals and buy-in from the community, which can create additional barriers to data collection. Food environment research is often conducted within pre-defined geographical boundaries; however, these boundaries do not always align with the spaces where people access food (39). Nevertheless, PM allows prioritization of food environment assessments to spaces that consumers most frequently engage thus is likely to yield more meaningful results in terms of characterizing food environment dimensions that directly influence communities’ food acquisition and purchase.

Another key learning from the PM that can help inform food environment research is just how important the natural and kin and community food environments (e.g., social networks) are in some settings. For example, if you were to solely conduct food environment assessments of the built environment, the researcher might be vastly misrepresenting the food that people have access to. In this Cambodia case study, this also would be true if mobile vendors were not included in the assessments given that they were one of the main sources of fresh food in several of the communes included in the study. Furthermore, the supplemental food acquired by sharing and exchange among community members would be missed, although found to be common practice among this study’s participants. For this reason, food environment research needs to include these food environment types. While measuring food accessed through the kin and community might be best done using surveys with consumers given that it would be difficult to observe these exchanges via food environment observations, observational assessments could be used to assess the food environment dimensions among mobile vendors. Findings from PM also offer the added value of interpreting the findings from food environment assessments conducted as well as consumer surveys.

By conceptualizing food environments in a more comprehensive way, from the perspective of the people living within a given community, we will be able to measure food environments in a way that more closely aligns with people’s lived experiences. PM provides a useful, easy to implement tool for conducting this with the view to better designing food environment research. This will be particularly important for communities that access different food environment types, such as those living in LMICs and indigenous communities. Aligning food environment assessments with people’s lived experiences can help to better characterize gaps in the availability, affordability, convenience, promotion and quality, or sustainability of food; however, there is a clear need for better methods and tools to measure these environments. By improving our ways of measuring food environments, we will be able to design better interventions that are more aligned to the needs of a given community.

## Data availability statement

The raw data supporting the conclusions of this article will be made available by the authors, without undue reservation.

## Ethics statement

The studies involving humans were approved by the Ethical approvals were obtained from the National Ethics Committee for Health Research in Cambodia and the Johns Hopkins University Homewood Institutional Review Board in the United States. The studies were conducted in accordance with the local legislation and institutional requirements.

## Author contributions

SD: Conceptualization, Data curation, Methodology, Supervision, Writing – original draft, Writing – review and editing. SM: Conceptualization, Data curation, Methodology, Supervision, Writing – original draft, Writing – review and editing. WS: Formal Analysis, Writing – review and editing. CK: Data curation, Writing – review and editing. SoS: Data curation, Methodology, Writing – review and editing. NC: Methodology, Writing – review and editing. JF: Conceptualization, Writing – review and editing. SeS: Conceptualization, Methodology, Supervision, Writing – review and editing.
