# Supplementary material for: Centering context when characterizing food environments: the potential of participatory mapping to inform food environment research
Source: Front Nutr. 2024 Feb 21;11:1324102. doi: 10.3389/fnut.2024.1324102 (PMC10914972; doi:10.3389/fnut.2024.1324102)

Supplementary Material

Centering context when characterizing food environments: the potential of participatory mapping to inform food environment research

Shauna Downs[1*], Swetha Manohar[2,3], Wiktoria Staromiejska[1], Chanvuthy Keo[4], Sophea Say[5], Nyda Chhinh[6], Jessica Fanzo[7], Serey Sok[8]

[1]Department of Health Behavior, Society and Policy, Rutgers School of Public Health, Newark, NJ, USA
[2]Global Food Ethics Policy Program, School of Advanced International Studies, 1776 Massachusetts Avenue, Washington DC, USA
[3]Nutrition, Diets and Health Unit, International Food Policy Research Institute, Washington DC, USA
[4]Faculty of Social Science and Humanities, Royal University of Phnom Penh, Russian Federation Boulevard, Khan Toul Kork, Phnom Penh, Cambodia
[5] Department of Tourism, Royal University of Phnom Penh, Phnom Penh 12150, Cambodia
[6]Department of Economic Development, Faculty of Development Studies, Royal University of Phnom Penh, Russian Federation Boulevard, Khan Toul Kork, Phnom Penh, Cambodia
[7]Columbia's Climate School, Columbia University, New York, NY, USA
[8]Research Office, Royal University of Phnom Penh, Russian Federation Boulevard, Khan Toul Kork, Phnom Penh, Cambodia

*** Correspondence:**
Shauna Downs
sd1081@sph.rutgers.edu

Keywords: food environment, participatory mapping, Mekong River, Tonle Sap Lake, focus group discussion

Supplementary Material A

River in Transition Study
FORM 1: Focus Group Discussion (FGD) Participatory Social Mapping Interview Guide

A. IDENTIFIERS

**Province:** ☐	1 = Prey Veng<br>2= Stung Treng<br>3 = Siem Reap<br>4 = Kampong Chhnang
**District [*srok/ khan*]:** ☐	1= Peam Chor<br>2= Preah Sdach<br>3= Siem Bouk<br>4= Thala Barviat<br>5= Prasat Bakong<br>6= Kampong Tralech
**Commune [*khum*]:** ☐	1= Kaoh Roka<br>2 = Preah Sdach<br>3= Siem Bouk<br>4= Chamkar Leu<br>5= Kampong Phluk<br>6 = Kantreang<br>7 = Kampong Tralech<br>8= Thma Edth
**FGD Location:** ____________________	Name of location (e.g. school, community center)
**Worker ID #1:** ☐☐ **Worker ID #2:** ☐☐	ID numbers of facilitator & research assistant
**Consent:** ☐ ☐	0 = No, ≥ 1 refused<br>1 = Yes, all consented
**Total # of women:** ☐☐	≥ Number of women
**Interview date:** ☐☐/☐☐/☐☐☐☐	Roman date
**Total time taken** ☐.☐	Hours

**Form status:** ☐	0 = Not complete<br>1 = Form complete

	**What is your age?**	**What is the highest level of education completed?**	**What is your main job?**	**What is your marital status?**	**How many children do you have?**
	≥18 = Years<br>99 = Don't know	0= None<br>1= Some primary, but not completed<br>2= Completed primary<br>3= Some secondary, but not completed<br>4= Completed secondary school<br>5= Some higher education but no degree<br>6= Completed higher education degree & more<br>9= Don't know	1= Take care of household<br>2= Small business owner<br>3= Daily/casual worker<br>4= Full-time salaried employment<br>5= Other (specify)<br>________<br>9= Don't know	1= Single<br>2= Married<br>3= Divorced<br>4= Widowed<br>5= Separated<br>6= Other (specify)<br>________<br>9= Don't know	≥ 0 = kids
Women #1	☐☐	☐	☐	☐	☐☐
Women #2	☐☐	☐	☐	☐	☐☐
Women #3	☐☐	☐	☐	☐	☐☐
Women #4	☐☐	☐	☐	☐	☐☐
Women #5	☐☐	☐	☐	☐	☐☐

Women #6	☐☐	☐	☐	☐	☐☐
Women #7	☐☐	☐	☐	☐	☐☐
Women #8	☐☐	☐	☐	☐	☐☐

Facilitator Instructions:

1. *Read the 'Study information overview" out loud to the group requesting the attention of participants and to ask questions if they have any. Answer and questions that arise.*
2. *Asks each participant to state their consent to participate in the FGD on the audio-recording device.*
3. *Inform participants that the FGD will last between 60-90 minutes.*
4. *Inform participant on the role of the Facilitator and the Note taker*
5. *Inform participant that refreshments will be served during the group discussion,*
6. *Confirm that socio-demographic information of each participant is collected upon arrival.*
7. *Share the social mapping process briefly.*
8. *Complete Parts 1 & 2*
9. *Thank participants for attending the FGD.*

B. PART 1: Characterizing the commune landscape

Facilitator Script [Read aloud]: For the first part of the discussion, we would like you to work as a group to draw a map of the places you go to get food within your commune. We will then talk about which foods you get from each of these places and the reason for getting them from that location. Would someone like to volunteer to start drawing the map of your community?

[once volunteer is identified] [Read aloud]: [NAME] will start drawing the map of your commune with input from the group. You can start by identifying a main road and any important landmarks. [DRAW MAIN ROADS AND LANDMARKS FOR COMMUNE].

C. PART 2: Identifying commonly used vendors in the commune

Facilitator Script: Now that we have the main roads and landmarks for the community drawn on the map, I want you to identify the different places that you, or people you know, get their food from. This includes the bodies of water, forests, plots, and gardens as well as markets. Add all of these places to the map. If the markets have names, I would like you to include these on the map as well. For the open air markets, I want you to label them as daily or weekly markets.

NOTE for facilitator*: The map does not have to be drawn to scale or to include every single vendor if there are a large number. The goal is to identify the main places that people purchase their food from and why they purchase these foods from those locatio*

NOTE for facilitator*: The following questions can be asked after the map is completed in Part 2 or during the Part 2 mapping process. Be sure to bring discussants back to the map when discussing different markets and sources of food. Map out places that come up later in the discussions – for example a forest or plot of land or ice cream cart.*

Facilitator Script [Read aloud]: First, I would like to ask you about the foods that you get from the market.

1. Which markets or vendors do you, and other people you know, purchase the most food from?

   i. Which foods do you purchase from these markets or vendors?
   ii. Why do you choose to purchase your food from these markets or vendors?
   PROBE: How do you access this market (e.g., bus, car, walking, etc.)? How much time does it take to travel from your home to the market?
   PROBE: How have these markets changed due to the COVID-19 pandemic?
   iii. How often do you purchase foods from [NAME MARKET/VENDOR]?
   iv. How do you get from your home to [NAME MARKET/VENDOR]?

2. [Read aloud] Which markets or vendors do you, and other people you know, purchase the least amount of food from?

   i. Why do you purchase less food from these markets or vendors?

3. [Read aloud] For each of the markets/vendors types (e.g., supermarkets, convenience stores, street vendors, etc) that are included on the map, ask the following questions:

   i. Which foods do you purchase from [NAME MARKET/VENDOR TYPE]?
   ii. Why do you purchase these specific foods from [NAME MARKET/VENDOR TYPE]?
   iii. How often do you purchase foods from [NAME MARKET/VENDOR TYPE]?
   iv. How do you get from your home to [NAME MARKET/VENDOR]?

[Read aloud] We will now take a break for some refreshments and continue discussing these markets you use commonly when you come back.

NOTE for faciliator*: Break for refreshements.*

4. [Read aloud] Have the markets/vendors that you access changed in recent years?

    i. Why have they changed?
    PROBE: How have these markets changed due to extreme climate events like flooding? How have these markets changed due to extreme climate events like flooding? How have these markets changed due to changes to the river (e.g. low water levels, overfishing/illegal fishing)?

Facilitator Script continued: Now I would like to ask you about the places you collect, fish, or grow food.

5. [Read aloud] Where are the places you and others in your commune grow food?

    i. Which foods are grown?
    ii. Who consumes these foods?
    iii. Do you exchange these foods with neighbors, friends or other people in the commune?

6. [Read aloud] Where are the places that you, and others in your commune, fish for food?

    i. What types of foods (fish and aquatic plants) are acquired?
    ii. Who consumes these foods?
    iii. Do you exchange these foods with neighbors, friends or other people in the commune?

7. [Read aloud] Where are all the places that you, and others in your commune, collect or forage for food?
    i. What types of foods are collected?
    ii. Who consumes these foods?
    iii. Do you exchange these foods with neighbors, friends or other people in the commune?

[Read aloud] That is the end of our questions. Before we finish, is there anything else that you want to share about where you get your food?

[Read aloud] Thank you for active participation.

Supplementary Materials B

A food environment map drawn by participants as part of a participatory mapping exercise during their focus group discussion. The participants resided in Thma Edth commune, Kampong Tralach district, Kampong Chhnang province, Cambodia.

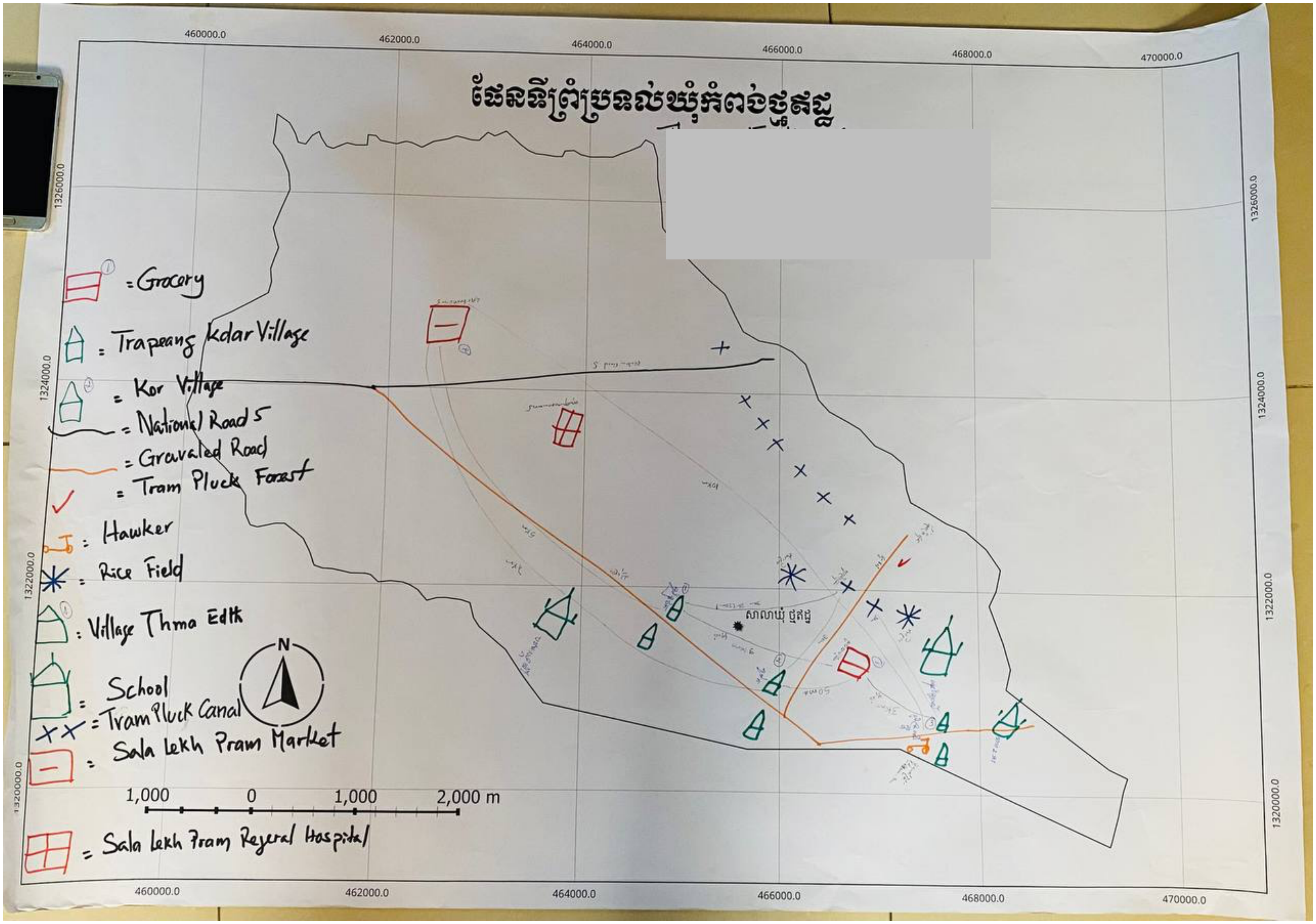

Supplement: Supplementary file 1 [file Data_Sheet_1.pdf]
